# Does Executive Function Influence Walking in Acutely Hospitalized Patients With Advanced Parkinson's Disease: A Quantitative Analysis

**DOI:** 10.3389/fneur.2022.852725

**Published:** 2022-07-19

**Authors:** Johanna Geritz, Julius Welzel, Clint Hansen, Corina Maetzler, Markus A. Hobert, Morad Elshehabi, Alexandra Sobczak, Jennifer Kudelka, Christopher Stiel, Johanne Hieke, Annekathrin Alpes, Nico Bunzeck, Walter Maetzler

**Affiliations:** ^1^Department of Neurology, University Hospital Schleswig-Holstein, Kiel, Germany; ^2^Department of Psychology and Center of Brain, Behavior and Metabolism (CBBM), University of Lübeck, Lübeck, Germany

**Keywords:** Parkinson's disease, straight walking, wearable sensors, executive functions, dual task, aged

## Abstract

**Introduction:**

It is well-known that, in Parkinson's disease (PD), executive function (EF) and motor deficits lead to reduced walking performance. As previous studies investigated mainly patients during the compensated phases of the disease, the aim of this study was to investigate the above associations in acutely hospitalized patients with PD.

**Methods:**

A total of seventy-four acutely hospitalized patients with PD were assessed with the delta Trail Making Test (ΔTMT, TMT-B minus TMT-A) and the Movement Disorder Society-revised version of the motor part of the Unified Parkinson's Disease Rating Scale (MDS-UPDRS III). Walking performance was assessed with wearable sensors under single (ST; fast and normal pace) and dual-task (DT; walking and checking boxes as the motor secondary task and walking and subtracting seven consecutively from a given three-digit number as the cognitive secondary task) conditions over 20 m. Multiple linear regression and Bayes factor BF_10_ were performed for each walking parameter and their dual-task costs while walking (DTC) as dependent variables and also included ΔTMT, MDS-UPDRS III, age, and gender.

**Results:**

Under ST, significant negative effects of the use of a walking aid and MDS-UPDRS III on gait speed and at a fast pace on the number of steps were observed. Moreover, depending on the pace, the use of a walking aid, age, and gender affected step time variability. Under walking-cognitive DT, a resolved variance of 23% was observed in the overall model for step time variability DTC, driven mainly by age (β = 0.26, *p* = 0.09). Under DT, no other significant effects could be observed. ΔTMT showed no significant associations with any of the walking conditions.

**Discussion:**

The results of this study suggest that, in acutely hospitalized patients with PD, reduced walking performance is mainly explained by the use of a walking aid, motor symptoms, age, and gender, and EF deficits surprisingly do not seem to play a significant role. However, these patients with PD should avoid walking-cognitive DT situations, as under this condition, especially step time variability, a parameter associated with the risk of falling in PD worsens.

## Introduction

Idiopathic Parkinson's disease (PD) is a neurodegenerative disorder characterized by specific motor symptoms, such as bradykinesia and rigidity, and several non-motor symptoms, such as cognitive impairment and depression (1, 2). The progression of these symptoms and the associated limitations, particularly deteriorated walking performance, can lead to reduced quality of life (3). Due to this progressive aggravation of both motor and non-motor symptoms accompanied by the effects of age as well as a history or risk of falls, patients with advanced PD may increasingly require inpatient medical treatment (4). However, these vulnerable patients are often not included in the studies (5, 6). Furthermore, the association between specific non-motor symptoms and walking performance in patients with PD is not fully understood. Hence, an important open question is how motor and specific non-motor symptoms are related in the advanced stage of the disease in acutely hospitalized patients.

Typical motor symptoms can be accompanied by reduced walking performance, i.e., decreased gait speed, increased asymmetry, and impaired rhythmicity and stability of gait (5). These symptoms lead to daily life-relevant limitations, especially concerning mobility. As motor impairments progress, the risk of falls increases and patients become more dependent (for example, being in need of using walking aids). Both factors are associated with reduced quality of life (4, 7). To detect motor impairment in PD, wearable devices have been increasingly used in recent years as a flexible and cost-effective option in clinical settings (7–10).

Among the non-motor symptoms in patients with PD, cognitive impairment, namely, deficits in cognitive flexibility, set shifting, and working memory (the so-called fronto-striatal associated executive functions, EFs), as well as in divided attention and keeping attentional focus, play an important role [reviewed in (23, 24)]. Even in the early stages of the disease and also in patients with PD without dementia, deficits in internal attentional control, cognitive flexibility, and planning actions have been reported (16). Cognitive impairment and dementia in PD are associated with an increased risk of falls (25) and reduced quality of life (6).

In everyday situations, walking is not merely a simple task but rather requires the ability to manage multiple tasks simultaneously. This complex process requires a high degree of cognitive flexibility and integration of movement sequences and external stimuli, depending on environmental demands. In light of this, recent studies have investigated a possible link between limited walking performance and deficits in EF and attention both in older healthy individuals and in patients with PD (12–15, 17, 22, 26–35). These studies typically examined walking under both single task (ST) and dual-task (DT) conditions, with different methods, paradigms, and outcome parameters. A meta-analysis showed negative associations between age and cognitive status, as well as age and gait speed under DT in healthy older adults (21). In addition, in a longitudinal study over 6 years with healthy older adults (*n* = 583, aged 65 and older), reduced cognitive flexibility [measured by the Trail Making Test, TMT (36)] was identified as a predictor for increasing mobility impairment and mortality (20). Another study found associations between poor TMT performance and changes in DT prioritization during walking at the expense of gait speed in older adults (11). Overall, the existing evidence suggests that healthy older adults under DT strategically adapt to increased demands, e.g., by reducing gait speed or requiring increased reaction time during cognitive tasks, but do not exhibit extensive changes in walking performance (32). In contrast, patients with PD appear to need higher levels of attention, executive control, and cognitive flexibility for actions such as walking. During the course of the disease, coping with increasing task complexity becomes more difficult for patients with PD (23, 32, 37–39). Comparative studies have shown that EF performance and associated walking impairment (primarily reduced gait speed and increased gait variability) are worse in patients with PD than in healthy controls, especially under DT conditions (15, 22). In addition, studies in patients with PD have shown that spatio-temporal walking parameters, such as gait speed and stride length, gait variability, and postural control, may be differently affected by impaired EF (14, 18, 34, 38, 40). These findings suggest that deficits in EF and divided attention in PD are associated with impaired walking performance and altered task prioritization as cognitive demands increase. The complexity of the (gait) situation is particularly evident with regard to higher dual-task costs (DTCs) while walking (14, 38, 41).

However, the studies mentioned above could not identify EF and divided attention as a relevant predictor for specific walking parameters and mainly focused on single walking parameters or used group comparisons or simple correlations (14, 34, 38). Cognitive impairment, advanced disease stage, severe motor symptoms, and needing a walking aid were the exclusion criteria (either combined or single) in most of the studies. Also, acutely hospitalized patients were often not included. However, these aspects are highly relevant for treatment indications, risks as well as quality of life, and patients' ability to cope with everyday life (5–7). Thus, it remains unclear to what extent EF and divided attention have an influence on specific aspects of gait for patients with advanced PD in acute need of inpatient care. Furthermore, many studies are conducted under laboratory conditions, which means that the results are not necessarily transferable to clinical diagnostics or the home environment (19). Further investigation focusing on the understanding and clinical considerations that follow from these findings is important. Therefore, the aim of this study was to investigate the association between EF, divided attention, and walking performance under ST and DT conditions in acutely hospitalized patients with advanced PD. We also included patients with severe symptoms (e.g., cognitive impairment and reduced walking performance). In doing so, different requirements under ST as well as under DT with both congruent (i.e., predominantly motor) and divergent (i.e., cognitive) additional demands during straight walking were investigated. Walking performance was assessed using spatio-temporal walking parameters to identify those associated with EF and divided attention in PD. Outcomes were measured with assessments integrated into the clinical routine on a neurogeriatric ward.

## Methods

This study was a part of the exploratory, observational multicenter study “COgnitive and Motor interaction in the Older populatioN” (ComOn). In the ComOn study, participants aged 50 years and older with at least one chronic disease are included. The main aim of the study was to gain a better understanding of the multifaceted symptoms of this cohort and their complex interactions using quantitative and digital parameters. Therefore, a comprehensive examination protocol to assess cognitive, motor, behavioral, and other clinical parameters was conducted. For the full examination protocol, we referred a previous study (42). The focus of these analyses is on the influence of EF and divided attention on straight walking performance in patients with advanced PD.

The data presented here were collected between October 2017 and November 2020 at the Department of Neurology, University Hospital Schleswig-Holstein Campus Kiel (Germany). Informed oral and written consent was obtained from all participants and, if necessary, their legal representative or assistance (e.g., due to cognitive impairment or dementia). The study was reviewed by the ethics committee of the Medical Faculty of the University of Kiel (ethics application number D 427/17).

### Participants

The study included geriatric inpatients diagnosed with PD (*n* = 119) according to the United Kingdom Parkinson's Disease Society Brain Bank Diagnostic Criteria (43) and the Movement Disorder Society (MDS) clinical diagnostic criteria for PD (44, 45). All participants fulfilled the inclusion criteria of the ComOn study protocol (42). Briefly summarized, participants were included if they were 50 years or older, able to walk at least 3 m independently with or without walking aid, and had sufficient hearing and visual acuity as well as sufficient speech comprehension as judged by the investigator. Main reasons for inpatient admission were deterioration in mobility and walking ability or general condition, recent falls, or medication adjustment due to reduced drug effects. Patients with severe motor symptoms measured by the MDS-revised motor part of the Unified Parkinson's Disease Rating Scale [MDS-UPDRS III, (46)] as well as patients with previously described mild cognitive impairment (MCI) or mild to moderate dementia were included (refer to Section Demographical and Clinical Parameters). Patients were excluded if they scored <5 points in the Montreal Cognitive Assessment [MoCA, (47)] as a cutoff value for severe dementia in PD (48). Patients with more than two falls in the past week were excluded due to safety reasons in the motor assessment.

### Procedure

Assessments took place in a clinical setting within the first 2 days after admission to the neurogeriatric ward. On the day of admission, a detailed medical history was conducted, and participants were given self-reporting questionnaires on various behavioral and clinical aspects. On the first day of treatment, a detailed neuropsychological examination was carried out, followed by a comprehensive movement analysis using inertial measurement units (IMUs, see Section IMU System). The duration of the two latter assessments was about 60 to 90 min each. Between the assessments, participants had a break of at least 60 min. The movement analysis was carried out on the ward corridor (>3 m broad, well-lit) in a designated area for this purpose. For this study, the data for straight walking over 20 m in ST and DT conditions were considered. To examine the patients in their best mobility condition possible, the medication was to be administered at a suitable time interval prior to the measurement after consulting with the medical staff.

### Measures

#### Demographical and Clinical Parameters

Age, gender, years of education [total number of years in school plus standard time period for any completed professional education (49)], and current disabilities (e.g., care level, frailty, vision, and hearing impairments and urinary incontinence) were collected *via* interview using geriatric screening tools which are described in detail in the ComOn study protocol (42, 50, 51). From the medical records, PD duration and aspects of previously described cognitive deficits were extracted. In addition, the MoCA was performed to assess global cognitive performance (47). Depressive symptoms were assessed using the screening questionnaire *Depression im Alter* Scale [DIA-S, (52)]. Based on the medication schedule at admission, the levodopa equivalent daily dose [LEDD (53)] was determined.

The MDS-UPDRS III (46) was used to evaluate the severity of motor symptoms. We scored values below 30 as mild, between 30 and 60 as moderate, and values above 60 as severe PD motor stage [adapted from (54)]. Moreover, the modified Hoehn & Yahr Scale (46) was assessed. Furthermore, the occurrence of dyskinesia (according to the MDS-UPDRS definition as involuntary, random movements) during the examination as well as their impact on the rating of the MDS-UPDRS III and the occurrence of freezing of gait (FOG) were recorded using the three related items of the MDS-UPDRS III (55, 56).

#### Executive Functions and Divided Attention

Executive function and divided attention were measured by the Trail Making Test (36). The TMT is a widely used neuropsychological paper-pencil test consisting of two parts, TMT A and TMT B (57). Both tasks captured the components of perceptual tracking as well as the processing speed. The TMT B also captured more complex executive functions such as alternating sequencing and set shifting (as a part of cognitive flexibility) and divided attention (57–59). In TMT A, circles with the numbers “1” to “25” must be connected as quickly as possible in ascending order. In TMT B, circles with the numbers “1” to “13” and the letters “A” to “L” must be connected alternately, again as quickly as possible. For both tasks, a test run with eight items was carried out in advance. The required time to complete each task was measured in seconds. Errors were corrected in a standardized way while time continued to run (57). In this study, the difference index ΔTMT (TMT B minus TMT A) was calculated. Several authors recommend using this derived score as it corrects for processing speed and therefore provides a better index of EF (11, 20, 59–62).

#### Straight Walking Performance

##### Walking Conditions

For the gait analysis, the participants were asked to walk a marked straight distance of 20 m four times. A different condition was set for each walk with increasing motor difficulty. During all four walks, participants wore an IMU system. It was documented whether patients completed the task with or without a walking aid. In condition one, *ST normal pace*, the distance was to be covered at a self-selected comfortable gait speed. In condition two, *ST fast pace*, participants were asked to walk as fast as possible without running. In condition three, *DT walking-cognitive*, participants were asked to subtract seven consecutively from a given three-digit number as fast as possible while walking at a fast pace. In condition four, *DT walking-motor*, predetermined boxes on a sheet of paper were to be crossed as quickly as possible with a pen while walking at a fast pace. Condition four was only possible for patients without a walking aid. Walking conditions were performed in the following order if patients had the capacity: ST fast pace, ST normal pace, DT walking-motor, DT walking-cognitive.

#### IMU System

Velcro straps were used to attach the RehaGait® IMU [Hasomed, Magdeburg, Germany (63)] to the patient's lower back at the level of the fifth lumbar vertebra before the gait assessment. The IMU is CE-certified and includes a triaxial accelerometer (±16 g) and a triaxial gyroscope (±2,000/s). Data were collected at a sampling frequency of 100 Hz and transmitted during the measurement *via* Bluetooth to a tablet with the RehaGait® application modified for the ComOn study in cooperation with the manufacturer.

#### Extraction and Analysis of Walking Parameters

Walking performance data were analyzed by an algorithm that has been validated for step detection in PD (64). From the raw data, the spatio-temporal parameters, number of steps and gait speed (m/s), double limb support time (DLS, s), mean step time asymmetry (ASYM, s; difference between mean step time difference between both feet), and step time variability (STV, s; square rooted sum of variance of step time for each foot divided by two) were calculated. A linear correction of DLS, ASYM, and STV to normalize for gait speed (to 1 m/s) was applied, as recommended in previous biomechanical studies on sensor-based walking parameters (41).

For the two DT conditions, the DTCs for walking (DTC_Walking_) were calculated for each of the parameters according to the formula *DTC* = (*ST*−*DT*)/*ST*× 100 (65), with positive DTC indicating deterioration of gait performance under DT compared to ST (31).

### Statistics

To address the question to which extent EF and divided attention are associated with quantitative walking parameters in PD, both multiple linear regression models and Bayesian regression models were calculated in all four walking conditions for each of the five walking parameters (number of steps, gait speed, DLS, ASYM, and STV) as well as their DTC_Walking_ in both DT conditions as outcome variables. Each model included ΔTMT as the predictor and MDS-UPDRS III, the use of a walking aid (except for DT walking-motor), age, and gender as covariates (using the forced entry method). Outliers, defined as ±3SD, were excluded. In detail, ΔTMT scores of two patients and DTC_Walking_ parameters of two patients (one in each of the two DT walking conditions) were excluded (Table 1). Model assumption multicollinearity (with variance inflation factor and tolerance), homoscedasticity, linearity and normality of residuals (with Q-Q-Plots), and independence of residuals (with Durbin-Watson) were checked (66). For the multiple linear regression models, the goodness of fit of each overall model using the R^2^_adj_ [adjusted for sample size n and multiple predictors using McNemar (66)] and the standardized regression weights β were determined and tested for significance (level of significance α < 0.05). *Post-hoc* Spearman's rho (ρ) correlation coefficient was calculated. For each Bayesian regression model, the Bayes factor BF_10_, as a measure for the strength of evidence in favor of one of two competing scientific theories (here, influence vs. no influence of EF and divided attention on walking performance) provided by the data (67, 68), was estimated using the Bayesian information criterion (BIC, (69). BF_10_ was classified, according to Lee and Wagenmakers (70), as follows: with BF_10_ above ten (for H1, here: EFs are associated with walking parameters) but below 0.03 (for H0, here: EFs are not associated with walking parameters) as “strong evidence” BF_10_ between three and ten (H1), respectively, of 0.10 and 0.03 (H0) as “moderate evidence” BF_10_ between one and three (H1), respectively, of 0.33 and 0.10 as “anecdotal evidence” (for H0), and BF_10_ = 1 as no evidence (70). Differences between the four walking conditions were calculated for ΔTMT, MDS-UPDRS III, age (using Kruskal–Wallis H test), and gender [using χ^2^ test, (66)]. As an additional explorative analysis, differences in ΔTMT, MDS-UPDRS III, age, and gender between patients with and without walking aid were calculated for the ST normal pace, the ST fast pace, and the DT walking-cognitive conditions [using the Mann–Whitney U test for continuous variables and Fisher's exact test for gender as dichotomous (66)].

**Table 1:** Descriptive characteristics of demographic, clinical, and walking parameters over all four walking conditions and of DTC_Walking_ over both DT walking conditions.

**demographic and clinical parameters**	**ST normal pace**		**ST fast pace**		**DT walking-cognitive**		**DT walking-motor**	
	***n***	**M (SD) [min; max] {Median; IQR}**	***n***	**M (SD) [min; max] {Median; IQR}**	***n***	**M (SD) [min; max] {Median; IQR}**	***n***	**M (SD) [min; max] {Median; IQR}**
age [years]	74	72 (8.39) [48;87] {75; 12}	60	73 (8.78) [48;83] {77; 12}	45	72 (9.55) [48;83] {77; 12}	34	71 (10.0) [48;81] {76.5; 14}
Women [*n* (%)]		25 (11)		17 (12)		12 (13)		7 (14)
Education [years]		10 (1.88) [6;14]		10 (1.79) [6; 14]		10 (1.89) [6;14]		10 (2.12) [6;14]
Disease duration [years]		10 (6.85) [0;25]		10 (7.02) [0;25]		9 (6.48) [0;24]		8 (5.68) [0;20]
Hoehn & Yahr		{3; 1}		{3; 1}		{3; 1}		{3; 0}
LEDD [mg]		748 (370.7) [100;1811]		717 (368.5) [100;1811]		707 (389.8) [100;1811]		648 (373.9) [100;1811]
MoCA		23 (3.27) [15;29]		23 (3.36) [15;29]		24 (3.30) [17;29]		23 (3.57) [15;29]
DIA-S		3 (2.3) [0;9]		2 (2.35) [0;9]		2 (2.47) [0;9]		2 (2.55) [0;9]
ΔTMT [s]		129 (81.5) [16;399] {104; 116}		128 (81.5) [16;399] {104; 88}		111 (78.2) [16;399] {83; 55}		125 (79.3) [16;303] {83.5; 116}
MDS-UPDRS III		30 (14.8) [4;60] {28.5; 25}		30 (14.4) [4;60] {28; 23}		28 (14.9) [4;60] {26; 23}		24 (14.0) [4;60] {22; 18}
Occurrence of dyskinesia [*n* (%)]		15 (14)		11 (15)		6 (16)		5 (17)
Impact of dyskinesia [*n* (%)]		5 (7)		3 (5)		2 (4)		0 (0)
Occurrence of FOG		29 (18)		25 (19)		15 (20)		9 (13)
Walking aid [*n* (%)]		23 (21)		17 (12)		11 (22)		0
**Walking parameters**								
Number of steps	74	41.2 (11.7) [20;80]	60	39.2 (11.6) [23; 77]	45	45.1 (16.8) [23; 104]	34	46.5 (17.8) [27; 106]
Gait speed		0.78 (0.21) [0.34;1.25]		0.98 (0.29) [0.38; 1.64]		0.74 (0.25) [0.26; 1.25]		0.76 (0.29) [0.29; 1.33]
DLS		0.37 (0.1) [0.14; 0.82]		0.39 (0.06) [0.3; 0.72]		0.4 (0.12) [0.23; 0.87]		0.38 (0.07) [0.22; 0.53]
ASYM		0.03 (0.05) [−0.05; 0.23]		0.04 (0.03) [−0.005; 0.16]		0.05 (0.04) [0.0006; 0.15]		0.04 (0.03) [0.004; 0.13]
STV		0.04 (0.05) [−0.04;0.2]		0.07 (0.04) [−0.02;0.2]		0.06 (0.05) [0.0001; 0.34]		0.06 (0.03) [0.02; 0.13]
**DTC**_**Walking**_								
DTC_Walking_ Number of Steps [%]					44	6.35 (20.6) [−84.0; 58.6]	33	13.4 (16.8) [−22.2; 43.2]
DTC_Walking_ gait speed [%]						8.14 (25.5) [−97.2; 61.2]		9.55 (33.3) [−136; 63.1]
DTC_Walking_ DLS [%]						4.22 (18.9) [−42.6; 54.7]		0.30 (13.8) [−30.9; 34.4]
DTC_Walking_ ASYM [%]						−0.41 (161) [−620; 348]		−40.1 (165) [−515; 209]
DTC_Walking_ STV [%]						46.9 (139) [−191; 589]		−5.11 (82.7) [−281; 129]

*ASYM, asymmetry; DIA-S, Depression im Alter Scale; DLS, double limb support; DT, dual task; DTC_Walking_, dual-task costs for walking while doing a second task (in percentage, %); FOG, Freezing of gait; IQR, interquartile range; LEDD, levodopa equivalence daily dose (in milligram, mg); M, mean; max, maximum; MDS-UPDRS III, Movement Disorder Society-revised version of the motor part of the Unified Parkinson's Disease Rating Scale; min, minimum; MoCA, Montreal Cognitive Assessment total score; n, sample size; s, seconds; SD, standard deviation; ST, single task; STV, step time variability; ΔTMT, delta of Trail Making Test (part B minus part A)*.

Data were preprocessed using MATLAB [version 2020b, (71)] and Python [version 3.9.1., (72)] Statistical analysis was conducted using JASP [version 0.14.1, (73)].

## Results

### Descriptive Characteristics

Out of the 119 (*n*) patients with PD who participated in the ComOn study and performed the TMT, a total of 74 participants with complete IMU-based data were included for this analysis (*n* = 45 did not perform the 20-m walking tasks due to the lack of capacity or motivation). In this overall group, the mean age was 72 years (SD = 8), 34% (*n* = 25) of participants were women, and the mean period of education was 10 years (SD = 2). Mean disease duration was 10 years (SD = 7), the median Hoehn & Yahr stage was 3 (IQR = 1), mean MDS-UPDRS III was 30 points (SD = 15), and mean LEDD was 748 mg (SD = 371). According to the medical records, cognitive impairment was previously reported in 17.7% of the cohort, of which 8.8% were diagnosed with dementia. The mean MoCA score was 23 points (SD = 3.2) and thus was below the diagnostic cutoff for MCI in PD [26 points, (74)]. The mean score of the DIA-S was three points (SD = 2.3) and thus below the cutoff for suspected depressive mood [≥4 points, (52)], with 23% of the patients showing a depressive mood.

Complete data on IMU-based walking measurement were available from 74 participants for ST normal pace, *n* = 60 for ST fast pace, *n* = 45 for DT walking-cognitive, and *n* = 34 for DT walking-motor. The decrease in sample size is due to the fact that not all subjects were capable of participating in every condition, which can be explained by the increasing demands per condition and the prioritized order of the tasks (e.g., due to reduced physical capacity not necessarily, all subjects who passed the ST normal pace condition could also perform ST fast pace, etc.). In general, over all four walking conditions, participants were comparable with respect to age (H = 0.72 (3), *p* = 0.87, Table 1), gender (χ^2^ = 2.13 (3), *p* = 0.55, Table 1), and ΔTMT performance (H = 2.18 (3), *p* = 0.54, Table 1). A walking aid was used by one-quarter (DT walking-cognitive) to one-third (ST normal pace) of the participants. Dyskinesia occurred during the measurement in 13% (DT walking-cognitive) to 21% (ST normal pace) and had an impact on the MDS-UPDRS III ratings in 0 (DT walking-motor) to 7% (ST normal pace). FOG occurred in 27 (DT walking-motor) to 42% (ST fast pace) of the participants during the MDS-UPDRS III examination. DT walking-motor was only feasible for participants who did not require a walking aid, as the checking box task while walking required the use of both arms. This group also had a lower MDS-UPDRS III score, but there was no significant difference between the walking conditions (H = 3.84 (3), *p* = 0.28). Table 1 provides descriptive characteristics across all four walking conditions.

**Figure 1:**
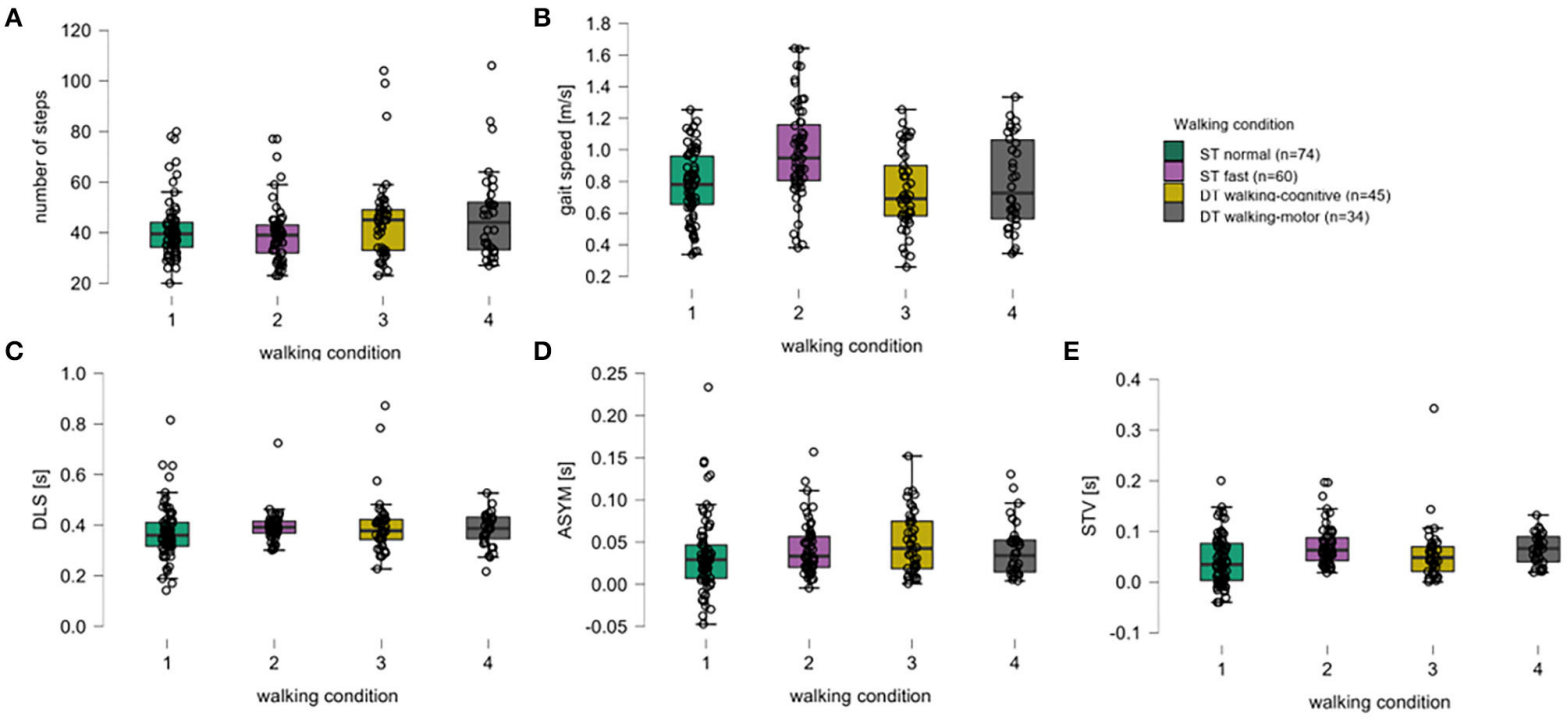
Box plots for all walking parameters over all walking conditions. For the five walking parameters, **(A)** number of steps, **(B)** gait speed (meters per second), **(C)** double limb support (DLS, seconds), **(D)** asymmetry (ASYM, seconds), and **(E)** step time variability (STV, seconds), the medians (thick black horizontal lines), the interquartile range (IQR, black-bordered boxes), and lower and upper whiskers (values within ±1.5xIQR), single-subject data points (black circles) are given for walking conditions of a single task normal pace (green, 1), a single task fast pace (violet, 2), the dual-task walking-cognitive (yellow, 3), and the dual-task walking-motor (gray, 4).

Concerning descriptive aspects of the walking parameters, the study participants used slightly fewer steps under ST conditions than under DT conditions. Under ST normal pace, the lowest values for mean DLS, mean ASYM, and mean STV were obtained. Under ST fast pace, participants had the lowest mean number of steps, walked the fastest on average, and showed the highest mean STV. Under DT walking-cognitive, they walked the slowest and had the highest mean DLS and mean ASYM (Figure 1, Table 1).

Under the DT walking-cognitive condition, higher DTCs were found for the number of steps, gait speed, DLS, and STV. For ASYM, DTCs were approximately zero. The highest DTCs were observed in the STV (46.9%). Under the DT walking-motor condition, higher DTCs were found in the parameters such as the number of steps and gait speed. Again, DLS did not show relevant DTC. Both ASYM (by about 40%) and STV (by about 5%) showed negative DTC (Table 1).

In the exploratory group comparison, patients who required a walking aid had significantly higher scores in the MDS-UPDRS III than patients without a walking aid in all three walking conditions (ST normal pace: W = 391, *p* = 0.02, ST fast pace: W = 244, *p* = 0. 005, DT walking-cognitive: W = 77, *p* = 0.004) as well as lower gait speed (ST normal pace: W = 901, *p* = 0.001, ST fast pace: W = 534.5*, p* = 0.006, DT walking-cognitive: W = 278, *p* = 0.02) and STV (St normal pace: W = 353, *p* = 0.004, ST fast pace: W = 575, *p* < 0.001, DT walking-cognitive: W = 297, *p* = 0.02). Under the DT walking-cognitive condition, patients with walking aid showed higher DTC_Walking_ at ASYM (median = 78.1 vs. median = 7.44, W = 69, *p* = 0.003) and STV (median = 108 vs. median = −3.88, W = 69, *p* = 0.003) than patients without walking aid. Under ST fast pace condition, patients with walking aid were older (median = 79 vs. median = 74, W = 215*, p* = 0.01) and took more steps (median = 42 vs. median = −36, W = 193.5, *p* = 0.005). There were no significant differences regarding ΔTMT, DLS, and ASYM and in gender distribution between these groups. Supplementary Table 1 provides detailed information.

### Regression Analyses

#### Single Task Walking Conditions

Under the ST normal pace condition, the overall multiple linear regression model with gait speed as the outcome parameter was significant (*p* = 0.002) with a coefficient of the determination of *R*^2^_adj_ = 24%. Therefore, the overall model, including age, gender, MDS-UPDRS III, walking aid, and ΔTMT, significantly explains 24% of gait speed variance. The effect was mainly driven by the use of a walking aid (β = −0.35, *p*=0.004) with a moderately negative *post-hoc* correlation (ρ = −0.43, *p* = 0.0001, **Figure 3A**) and, to less extent, by the MDS-UPDRS III (β = −0.21, *p* = 0.06) with a moderately negative *post-hoc* correlation (ρ = −0.32, *p* = 0.005, **Figure 3A**). ΔTMT (β = 0.02, *p* = 0.89), age (β = −0.12, *p* = 0.32), and gender (β = 0.04, *p* = 0.71) had no significant effect in the model. Also, the overall multiple regression model for STV was significant (*p* = 0.04) with *R*^2^adj = 16%. Here, the effect was again mainly driven by the use of a walking aid (β = −0.25, *p* = 0.05) with a moderately negative *post-hoc* correlation (ρ = −0.34, *p* = 0.003, **Figure 3B**) and, to less extent, by age (β = −0.12, *p*=0.09) with a low negative *post-hoc* correlation (ρ = −0.27, *p* = 0.09, **Figure 3B**). Despite the parametric regression models being significant, the Bayesian regression suggested moderate evidence for H0, indicating no relevant association of ΔTMT with neither gait speed (BF_10_ = 0.12) nor STV (BF_10_ = 0.13). Similarly, the Bayesian regressions provided moderate evidence for H0 with regard to the number of steps (BF_10_ = 0.13), DLS (BF_10_ = 0.13), and anecdotal evidence for H0 for ASYM (BF_10_ = 0.36). Therefore, individually significant effects were not further interpreted. Table 2 provides detailed information on the significant multiple regression models.

**Table 2:** Multiple linear regression models and Bayes factors for significant walking parameters and their DTC _Walking_.

	**Gait Speed**					**STV**					**Number of steps**				
	**ST normal pace (*****n*** **=** **74)**														
**Walking parameters**	**R^2^_**adj.**_**	**F**	**BF_**10**_**	**β**	**p**	**R^2^_**adj.**_**	**F**	**BF_**10**_**	**β**	**p**					
	0.24	4.31	0.12^a^		0.002^**^	0.16	2.55	0.13^a^		0.04^*^					
Age				−0.12	0.32				−0.12	0.09					
Gender				−0.04	0.71				−0.13	0.25					
MDS-UPDRS III				−0.21	0.06				0.04	0.73					
Walking aid				−0.35	0.004^**^				−0.25	0.05^*^					
ΔTMT				0.02	0.89				−0.06	0.63					
	**ST fast pace (*****n*** **=** **60)**														
	***R*** ^2^_**adj.**_	**F**	**BF**_**10**_	**β**	***p***	***R*** ^2^_**adj.**_	**F**	**BF**_**10**_	**β**	***p***	***R*** ^2^_**adj.**_	**F**	**BF**_**10**_	**β**	***p***
	0.22	3.10	0.14^a^		0.02^*^	0.18	3.51	0.13^a^		0.008^**^	0.19	2.60	0.14^a^		0.04^*^
Age				−0.17	0.21				−0.17	0.21				0.17	0.22
Gender				0.05	0.69				−0.24	0.06				−0.04	0.75
MDS-UPDRS III				−0.27	0.04^*^				0.09	0.45				0.25	0.06
Walking aid				−0.27	0.06				−0.30	0.03^*^				0.24	0.09
ΔTMT				0.05	0.72				−0.02	0.87				−0.04	0.76
**DTC**_**Walking−cognitive**_ **[%] (*****n*** **=** **44)**	**STV [%]**														
	***R*** ^2^_**adj.**_	**F**	**BF**_**10**_	**β**	***p***										
	0.23	3.50	0.18^a^		0.01^**^										
Age				0.26	0.09										
Gender				0.20	0.16										
MDS-UPDRS III				0.18	0.25										
Walking aid				0.26	0.12										
ΔTMT				0.08	0.58										

a *moderate evidence for H0; BF_10_, Bayes factor as a measure of strength of model evidence; β, standardized regression weights; DT, dual-task; DTC_Walking_, dual-task costs for walking while doing a second task (in percentage, %); F, test statistic from ANOVA used for testing significance of the multiple regression models; MDS-UPDRS III, Movement Disorder Society-revised version of the motor part of the Unified Parkinson's Disease Rating Scale; m/s, meter per seconds; n, sample size; p ≤ 0.05*

* *, significant on level of significance α ≤ 0.05; p ≤ 0.01*

** *, significant on level of significance α ≤ 0.01; R^2^_adj._, multiple regression coefficients adjusted for sample size; s, seconds; ST, single task; STV, step time variability; ΔTMT, delta of Trail Making Test (part B minus part A)*.

**Figure 2:**
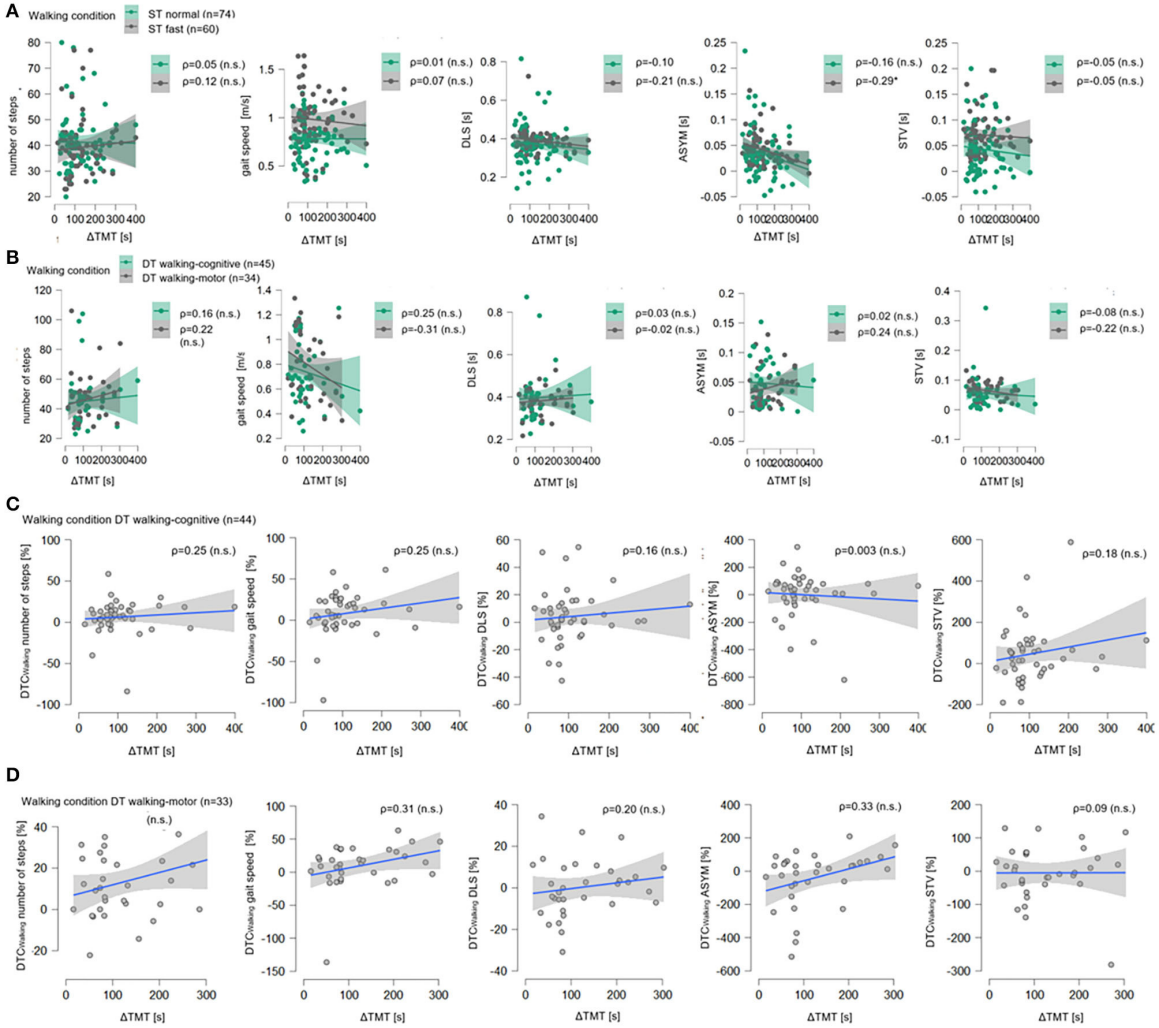
Correlation plots for ΔTMT with all walking parameters. In **(A)**, for the single-task normal pace walking condition (ST Normal, green) and the single-task fast pace condition (ST Fast, gray), all five walking parameters, i.e., number of steps, gait speed (in meter per seconds, m/s), double limb support (DLS), asymmetry (ASYM), and step time variability in seconds (STV, s) are shown on the ordinates, the delta of Trail Making Test (part B minus part A, ΔTMT) is on the abscissas. Sample size N is given as well as Spearman's rank correlation (ρ) between ΔTMT and each walking parameter, significant correlation coefficients are marked with ^*^ (level of significance p ≤ 0.05), non-significant ones are marked with (n.s). For each condition, data points (dots) and regression lines with confidence intervals (lines with surrounding boxes) are shown. In **(B)** the same is shown for the dual-task motor-cognitive walking condition (DT motor-cognitive, green) and the dual-task motor-motor condition (DT motor-motor, gray). In **(C)**, for DT motor-cognitive walking condition the dual-task costs while walking in percentage (DTC_Walking_, %) for all five walking parameters are shown on the ordinates, the delta of Trail Making Test (part B minus part A, ΔTMT) are on the abscissas as well as Spearman's rank correlation (ρ) between ΔTMT and each DTC_Walking_. Data points (gray dots) and regression lines with confidence intervals (blue lines with surrounding gray boxes) are shown for each parameter. The same is shown in **(D)** for the DT motor-motor walking condition.

**Figure 3:**
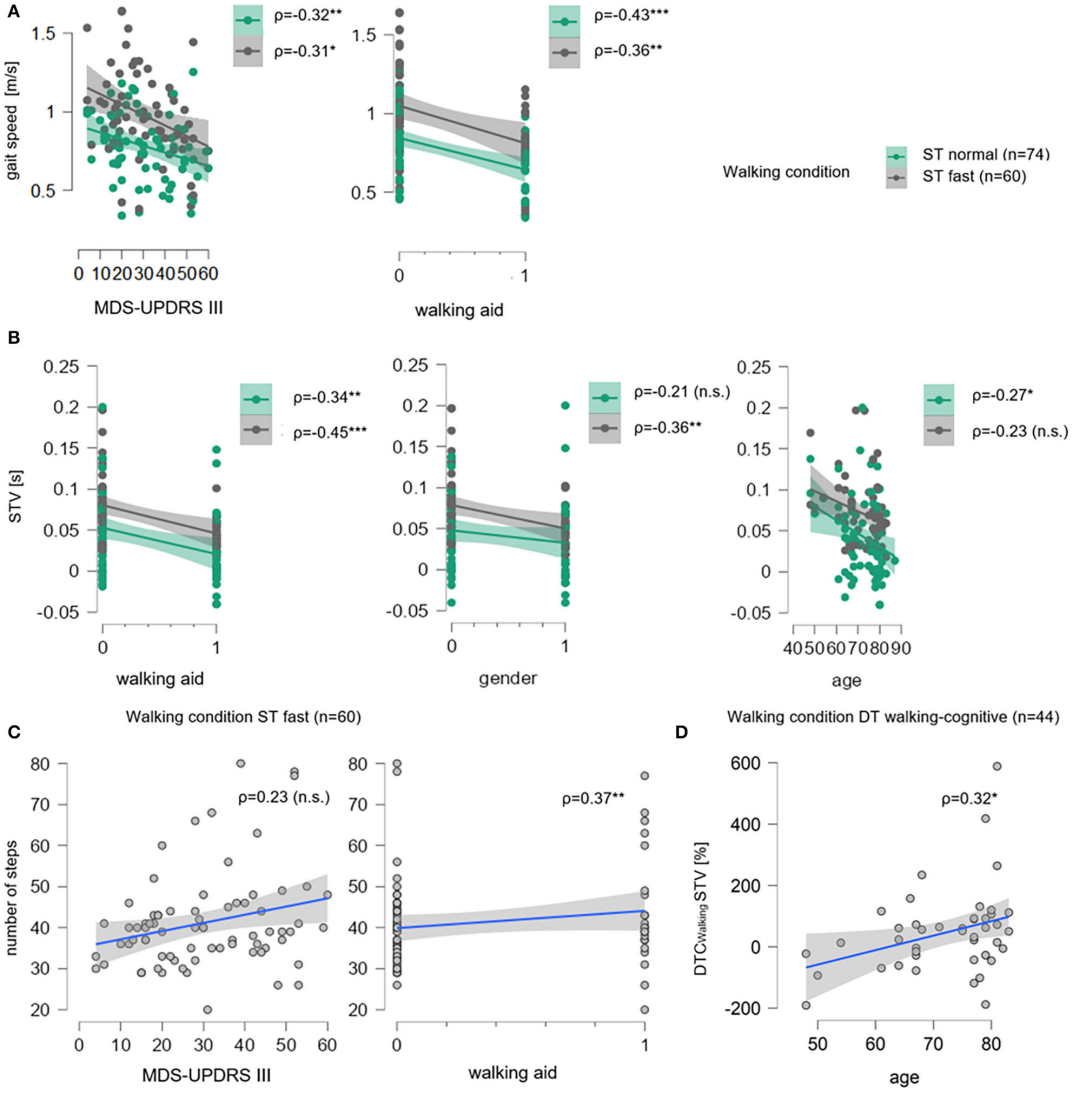
Correlation plots for significant multiple linear regression models for gait speed, STV, number of steps and DTC_Walking_ for STV. In **(A)**, for single task normal pace walking condition (ST Normal, green) and single-task fast pace condition (ST Fast, gray), gait speed (in meter per seconds, m/s) is shown on the ordinates, the total score of the Movement Disorder Society-revised version of the motor part of the Unified Parkinson's Disease Rating Scale (MDS-UPDRS III) and walking aid (0 = “no walking aid,” 1 = “walking aid”) are on the abscissas. Sample size n is given as well as Spearman's rank correlation (ρ) between gait speed and each parameter. For each condition, data points (dots) and regression lines with confidence intervals (lines with surrounding boxes) are shown, and significant correlation coefficients are marked with ^*^ (level of significance p ≤ 0.05), ^**^ (level of significance p ≤ 0.01), and ^***^ (level of significance p ≤ 0.001), non-significant ones are marked with (n.s.). In **(B)**, the same is shown for step time variability (STV in seconds, s) on the ordinates, and walking aid, gender (0= “men,” 1= “women”), and age on the abscissas. In **(C)** for ST fast pace number of steps is shown on the ordinates, MDS-UPDRS III and walking aid are on the abscissas. Here, data points (gray dots) and regression lines with confidence intervals (blue lines with surrounding gray boxes) are given for each parameter. In **(D)**, the same is shown for DT walking-cognitive walking condition for dual-task costs while walking in percentage (DTC_Walking_, %) of STV on the ordinate and age on the abscissa.

For the ST fast pace, the multiple linear regression model for gait speed was significant (*p* = 0.02, Table 2), with a variance resolution of *R*^2^_adj_ = 22%, driven by the MDS-UPDRS III (β = −0.27, *p* = 0.04) with a moderately negative *post-hoc* correlation (ρ = −0.31, *p* = 0.02, **Figure 3A**) and a negative trend for use of a walking aid (β = −0.27, *p* = 0.06) with a moderate negative *post-hoc* correlation (ρ = −0.36, *p* = 0.004, **Figure 3A**). For STV, the overall model was also significant (*p* = 0.008) with a variance resolution of *R*^2^_adj_ = 18%. Here, the model was driven by the use of a walking aid (β = −0.30, *p* = 0.03) with lower STV in patients without walking aid compared to patients with a walking aid, with a moderately negative *post-hoc* correlation (ρ = −0.45, *p* = 0.0003, **Figure 3B**), and a trend toward significance in the gender parameter with lower STV in women compared to men (β = −0.24, *p* = 0.06), with a moderately negative *post-hoc* correlation (ρ = −0.36, *p* = 0.005, **Figure 3B**). For the number of steps, the overall model was also significant (*p* = 0.04, Table 2) with a variance resolution of *R*^2^_adj_ = 19%, with no significant effect of a single predictor but trends toward significance for the use of a walking aid (β = −0.24, *p* = 0.09), with a moderately negative *post-hoc* correlation (ρ = −0.37, *p* = 0.004, **Figure 3C**) and the MDS-UPDRS III (β = −0.25, *p* = 0.06) with no significant *post-hoc* correlation (ρ = −0.23, *p* = 0.08, **Figure 3C**). Similarly, in the Bayesian regressions, there was moderate evidence for H0 for gait speed (BF_10_ = 0.14), STV (BF_10_ = 0.13), number of steps (BF_10_ = 0.14), and DLS. For DLS (BF_10_ = 0.23), there were no significant effects for the overall model of the multiple linear regression analyses. There was no significant effect for ΔTMT in any of the models. However, there was a significant negative correlation with ASYM (ρ = −0.29, *p* = 0.03, refer to Figure 2A), but Bayesian Regression again indicated anecdotal evidence for H0 for ASYM (BF_10_ = 0.40, no relevant association with gait speed with the ΔTMT included in the model).

#### Dual-Task Walking Conditions

For both DT conditions, there were no significant effects in any of the multiple linear regression models. There was no significant effect for ΔTMT in any of the models (Figure 2B). In the Bayesian regressions, there was moderate evidence for H0 under DT walking-cognitive for all walking parameters (BF_10_ between 0.15 and 0.19). The same was true for the multiple linear regression and Bayesian regression models, including ΔTMT, MDS-UPDRS III, age, and gender, but not for the use of a walking aid under DT walking-motor for the number of steps (BF_10_ = 0.19), gait speed (BF_10_ = 0.19), and STV (BF_10_ = 0.26). For ASYM (BF_10_ = 0.37) and DBL (BF_10_ = 0.36), however, there was again anecdotal evidence for H0.

#### Dual-Task Costs

With the cognitive task added, the overall multiple linear regression model was significant for DTC_Walking_ of STV (*p* = 0.01, Table 2) with a resolved variance of *R*^2^_adj_ = 23%, driven by a trend for age (β = 0.26, *p* = 0.09) with a significant moderately positive *post-hoc* correlation (ρ = 0.32, *p* = 0.04, Figure 3D). There were no further significant results for DTC_Walking_ of any other walking parameter.

With the motor task added, there were no significant results for DTC_Walking_ in the multiple linear regression models. Under both DT walking conditions, ΔTMT did not show a significant association with DTC_Walking_ in any of the regression models or significant correlations (Figure 2D). In the Bayesian regression, there was moderate evidence for H0 for all DTC_Walking_ under DT walking-cognitive conditions (BF_10_ between 0.16 and 0.31) as well as for all DTC_Walking_ of all walking parameters except ASYM (where there was again anecdotal evidence for H0, BF_10_ = 0.83) under DT walking-motor condition (BF_10_ between 0.19 and 0.26).

Figure 2 illustrates the results using correlation plots for all walking parameters with the ΔTMT for both ST conditions (A) and DT conditions (B) as well as for all DTC_Walking_ under both DT conditions [(C), (D)]. As for the walking parameters, the flat slopes of the regression lines and the wide dispersion of the data points suggest a lack of linear associations between ΔTMT and any of the walking parameters in all four conditions or the DTC_Walking_ in both DT conditions. *Post-hoc* Spearman's ρ correlation coefficients were also reported. Other than the abovementioned low negative correlation with ASYM under ST fast pace, there were no significant correlations to be found between the ΔTMT and any of the other walking parameters nor their DTC_Walking_.

Figure 3 illustrates the effects of the MDS-UPDRS III total score and the use of a walking aid on gait speed (A) and on STV (B) in the multiple linear regression models using correlations plots for both ST conditions. The slope of the regression degrees and the condensed location of the data points indicate a linear relationship between gait speed and both predictors. The trends of the MDS-UPDRS III total score and the use of a walking aid on a number of steps are similarly seen (C) as well as the age trend on DTC_Walking_ of STV under DT walking-cognitive condition (D).

## Discussion

The aim of this study was to investigate to which extent EF and divided attention (measured by ΔTMT performance) are related to specific aspects of walking performance in acutely hospitalized participants with advanced PD under ST and DT walking conditions. To our best knowledge, this is the first study to analyze several IMU-based spatio-temporal walking parameters and their DTC in this vulnerable cohort. Other studies either focused on single walking parameters (14), excluded patients with advanced PD and cognitive impairment (14, 22, 33, 34, 38), used different statistical methods [e.g., only correlation analyses (40)], or calculated group comparisons for ST and DT conditions (35), which addresses different scientific questions. Our results suggest that, especially, the severity of motor symptoms, the use of a walking aid, age, and gender have a relevant influence on walking performance in these patients. Concerning specific walking parameters, especially, gait speed and STV were significantly influenced, mainly under ST conditions. Furthermore, with an added cognitive task, increasing DTC_Walking_ of STV was also significantly influenced. Surprisingly, EF and divided attention do not seem to play a significant role.

Although some previous studies were able to reveal correlative and predictive relationships between EF and walking parameters, in this study, TMT performance has no significant predictor of specific spatio-temporal walking parameters, neither under ST nor under DT conditions. In one study that compared moderately affected patients with PD and healthy controls under ST and various DT walking conditions, EF performance was correlated significantly with gait variability (15). The authors concluded that gait variability and rhythmicity represent automated processes in healthy older adults but are more attention-demanding for patients with PD in the context of EF deficits in complex walking situations. In addition, studies have shown that different walking performance factors, such as spatio-temporal control, postural control, and variability, underlie different mechanisms that may also be differently affected by EF deficits in PD (18). For example, there is evidence that gait speed and stride length correlate positively with cognitive processing speed, whereas step width variability correlates positively with EF and attentional functions [as a calculated factor out of several cognitive tasks, (34)]. Also, patients with PD with poorer EF showed higher DTC, with EF accounting for 5% of the total DTC for gait speed and being identified as the best predictor of DTC (14), along with motor symptoms. However, compared with the results presented here, the authors could not uncover a significant relationship among EF, divided attention, and gait speed in any of the walking conditions. EFs were assessed with a different paradigm than the TMT and the walking distance was shorter, which would explain the different results in our study. In another study of advanced patients with PD (suffering from motor fluctuations) using comparable walking conditions and secondary tasks to this study, EF performance (measured by ΔTMT) was identified as a relevant predictor of DTC of gait speed (38). However, there were also differences in the methodology and characteristics of the subjects, which would explain the different results in our study. The walking distance was also four times longer than usual in our study and DTCs were calculated differently. Furthermore, participants were on average 8 years younger than the participants reported here, showed less severe motor symptoms (mean MDS-UPDRS III total score was 11 points lower), did not use a walking aid, and did not suffer from clinically relevant cognitive impairment. Our results match with the findings of another study on patients with advanced PD without cognitive impairment (40). The duration of a 3 m Timed-Up and Go Test (TUG) and EF (also measured by the TMT) was correlated moderately under both ST and DT, but TMT performance was not a significant predictor. This data and our findings suggest that performance in EF and divided attention tasks may not necessarily be linked linearly to common spatio-temporal walking parameters, such as gait speed, of these patients. Rather, the severity of PD-specific motor symptoms seems true to inflict the walking performance in this and other PD cohorts. Specifically, under ST, the increase in motor symptoms explains a decrease in spatio-temporal walking parameters, e.g., gait speed, which is also consistent with previous studies (14, 75). Moreover, our results suggest that patients with a walking aid are more affected by the underlying disease. Hence, being in need of a walking aid, which can be seen as an indicator of vulnerability, is another relevant factor with regard to a better understanding of deficits in walking performance in patients with advanced PD. Therefore, these factors should be prioritized regarding the diagnostics and treatment of walking performance deficits in an acute neurogeriatric setting.

Nevertheless, our results also provide evidence that cognitive aspects should not be disregarded in this vulnerable cohort. This may be particularly relevant for patients with dementia. Consistent with the literature, the results shown here indicate that patients with advanced PD show partly high costs in spatio-temporal walking parameters in situations where an additional demand is placed on them (14, 38). Depending on the secondary task type (convergent vs. divergent, i.e., walking-cognitive, respectively walking-motor) and motor difficulty, the costs vary (38). In the study presented here, DTCs are most pronounced in STV. Patients in both divergent (walking-cognitive) and convergent (walking-motor) DT conditions exhibited increased DTC_Walking_ for the number of steps and gait speed but not necessarily for DLS, STV, and ASYM. These findings fit with a previous study showing that, during walking under DT, patients with PD exhibited reduced gait speed and stride frequency compared to healthy controls (22). Interestingly, for the number of steps and gait speed, DTC_Walking_ tends to be higher in the convergent condition. This suggests that accomplishing another motor task while walking might require a higher level of brain capacity in similar areas and thus be more demanding on speed than an additional divergent task. In contrast to that, the highest DTC_Walking_ was found for STV (47%) in the divergent condition. For DTC_Walking_ of STV, the overall model explained about 23% of the variance. This suggests that, in this cohort, step time variability decreases when older patients with advanced PD are demanded to split attention between walking and a demanding cognitive task. This is also in line with a previous study (15), in which it was detected that gait variability was impaired under DT only in the PD group. Together, this suggests that gait variability needs to be brought into clinical focus as a diagnostic parameter, especially when assessing the ability to cope with more complex walking situations. This is particularly relevant given that increased STV is associated with falls in patients with PD (4, 8). Therefore, these patients should avoid those situations. This also can result in possible new implications for multimodal therapeutic interventions with regard to the trainability of STV under DT walking-cognitive condition. Interestingly, ASYM proved to be 40% better under DT than under ST in the presence of an additional convergent (i.e., predominantly motor) task. A possible explanation could lie in the specific execution of the motor task using a clipboard. The carrying of the clipboard and the demanded visual focus on the clipboard while checking boxes during walking could contribute to the compensation in the asymmetric walking performance. In addition, checking boxes themselves, as an external rhythm generator, could support step time symmetry. However, this requires further investigation on the underlying pathophysiological mechanisms. Nonetheless, if this is true, it might be relevant with regard to clinical diagnostics as well as the design of multimodal interventions, where this specific kind of additional task could be promising in the training of symmetrical walking. In addition, for the DT walking-motor condition, only patients without walking aid could be considered by definition. This makes comparability with the other three walking conditions (which included also patients with walking aids) difficult. Future studies should further focus on this aspect, using other secondary motor tasks that can also be performed with a walking aid. However, we believe that our study provides new insight regarding important factors influencing walking performance in acutely hospitalized patients with advanced PD. As so far there has been a lack of knowledge regarding this cohort that deserves special attention due to its vulnerability, our results contribute to the optimization of diagnostics and treatment in the neurogeriatric setting.

### Limitations

First, the influence of acute factors (e.g., infections, worsening of PD or other symptoms, and recent fall events) on the overall condition of the patients cannot be completely ignored. However, we argue that, as this group of patients requires special attention in treatment due to their health condition, a specific investigation of this cohort is justified. Second, the number of participants decreased successively with increasing the motoric task difficulty. Therefore, data of more severely affected patients are not included in the more complex gait tasks. Furthermore, randomization of the tasks was not possible for reasons of feasibility and to reduce errors in performance, as they were integrated into a comprehensive movement protocol [more detailed information provides (42)]. The decrease in the number of subjects in successful task performance with higher cognitive and motor complexity can be taken as an additional indication that these demands, as required in everyday life, can be increasingly poorly mastered by patients with advanced PD. Third, the tasks were adapted to the individual coping ability (with or without a walking aid) of the patients. This was done with the rationale of realistically representing a neurogeriatric PD cohort and achieving the most meaningful sample size possible. Fourth, patients were tested during the “ON” phase to collect data on the patients' best possible condition. Therefore, we cannot draw any conclusions regarding the un-medicated (“OFF”) status. Fifth, cognitive flexibility and divided attention were assessed with a previously established paradigm (TMT), which, however, only measures specific aspects of EF and attentional processes. It was selected because the purpose of the study was to detect associations using clinically established, well-validated (refer to Method section) and economically feasible methods. Sixth, due to the small sample size of this pilot, a more granular analysis of the severity of dyskinesia, the influence of freezing of gait or walking aids was not possible. Future studies with larger cohorts should focus on these aspects specifically for patients with advanced PD. Finally, our sample did not include healthy controls nor age-matched in-patients with other diseases as controls, which would allow more direct conclusions regarding pathology-specific aspects and to correct for effects of age and acute illness.

## Conclusion

To our knowledge, this is the first study to predict spatio-temporal walking parameters in acutely hospitalized patients with advanced PD. Therefore, these results provide new insights regarding walking performances in situations where an additional demand is placed on. A relevant predictive value of EF and divided attention for deficits in walking performance cannot be inferred from our study. However, our analyses provide evidence that more severe motor symptoms, being in need of a walking aid (and age), are associated with a reduced gait speed and higher STV, especially under ST conditions as well as with increasing DTC_Walking_ in STV when an additional cognitive task requires to split attention. Thus, for clinical diagnostics and treatment in an acute neurogeriatric setting, it remains essential to consider clinical symptoms. Furthermore, potential cognitive influences under DT walking situations, which can pose limitations and hazards (such as increased falls due to distraction), also need to be taken into consideration when evaluating new assessment methods for walking performances such as IMU data. Future studies should investigate to what extent deficits in EF and attentional functions may influence the benefit of therapeutic interventions for patients with PD in acute need of hospital care.

## Data Availability Statement

The original contributions presented in the study are included in the article/Supplementary Material, further inquiries can be directed to the corresponding author.

## Ethics Statement

The studies involving human participants were reviewed and approved by Ethics Commitee of the Medical Faculty of the Christian-Albrechts-University of Kiel. The patients/participants and, if necessary, their legal representative or assistance provided their written informed consent to participate in this study.

## Author Contributions

JG and WM made substantial contributions to the conception and design of all parts of the study, trained and supervised the examiners, were responsible for acquisition, analysis, and interpretation of data and in drafting as well as revising the manuscript. JW, CH, MH, JK, and ME made substantial contributions to the conception and design and training and supervision of the examiners. NB and AS made substantial contributions to the conception and design, analysis, and interpretation of the data. CM together with JG, JW, CH, and JK was responsible for the implementation of the database and organization of data. MH, ME, JK, AA, CS, and JH made substantial contributions to the conception and design regarding clinical data for their involved Department of Neurology in Kiel (Germany). All authors revised the manuscript critically for important intellectual content, given their final approval of the version to be published, and has participated sufficiently in the work and takes public responsibility for appropriate portions of the content and agrees to be accountable for all aspects of the work in ensuring that questions related to the accuracy or integrity of any part of the work are appropriately investigated and resolved.

## Funding

This study was funded by the Open Access Publication fund of the Christian-Albrechts-University of Kiel for junior scientists of the CAU by Schleswig-Holstein state government. We acknowledge the financial support by DFG within the funding programme Open Access-Publikationskosten.

## Conflict of Interest

The authors declare that the research was conducted in the absence of any commercial or financial relationships that could be construed as a potential conflict of interest.

## Publisher's Note

All claims expressed in this article are solely those of the authors and do not necessarily represent those of their affiliated organizations, or those of the publisher, the editors and the reviewers. Any product that may be evaluated in this article, or claim that may be made by its manufacturer, is not guaranteed or endorsed by the publisher.

## Abbreviations

ASYM: Mean step time asymmetry
ComOn: Cognitive and Motor Interaction in the Older Population
DIA-S: *Depression im Alter* Scale
DLS: Mean double limb support
DT: Dual task
DTC_Walking_: Dual-task costs while walking
FOG: Freezing of gait
LEDD: Levodopa equivalence daily dose
MCI: Mild Cognitive Impairment
MoCA: Montreal Cognitive Assessment
PD: Parkinson's disease
ST: Single task
STV: Mean step time variability
TMT: Trail Making Test
MDS UPDRS: Revised version of the Unified Parkinson's Disease Rating Scale.
