# Supplementary material for: Does Executive Function Influence Walking in Acutely Hospitalized Patients With Advanced Parkinson's Disease: A Quantitative Analysis
Source: Front Neurol. 2022 Jul 19;13:852725. doi: 10.3389/fneur.2022.852725 (PMC9344922; doi:10.3389/fneur.2022.852725)
Supplement: Supplementary file 1 [file Table_1.DOCX]

***Suppl.-Tab. 1: Explorative group comparison between patients with and without walking aid regarding parameters relevant for the regression models for the walking conditions ST normal pace, ST fast pace and DT walking-cognitive***

|  | **ST normal pace** | | | **ST fast pace** | | | **DT walking-cognitive** | | |
| --- | --- | --- | --- | --- | --- | --- | --- | --- | --- |
|  | Median (IQR) | | W^a^; p^a,b^ | Median (IQR) | | W^a^; p^a,b^ | Median (IQR) | | W^a^; p^a,b^ |
| walking aid group | with | without |  | with | without |  | with | without |  |
| n | 23 | 51 |  | 17 | 43 | ^b^ | 11 | 34 | ^b^ |
| age [years] | 77 (10) | 74 (12) | 435; 0.08 | 79 (5) | 74 (12.5) | 215; 0.01* | 77 (11.5) | 77 (14.25) | 154; 0.39 |
| female [n (%)] | 10 (44) | 15 (29) | 0.29^b^ | 8 (47) | 9 (21) | 0.66^b^ | 5 (46) | 7 (21) | 0.13^b^ |
| ΔTMT [s] | 103 (81) | 109 (129) | 604; 0.84 | 105 (54) | 103 (109) | 338; 0.66 | 94 (29.5) | 82.5 (65.5) | 169; 0.67 |
| MDS-UPDRS III | 37 (23) | 26 (22) | 391; 0.02* | 37 (18( | 25 (20) | 244; 0.05* | 42 (17.5) | 21 (16.5) | 77; 0.004** |
| number of Steps | 40 (11.5) | 39 (11) | 480; 0.22 | 42 (7) | 36 (12) | 193.5; 0.005** | 47 (5) | 41.5 (17) | 116; 0.06a |
| gait speed | 0.68 (0.24) | 0.83 (0.29) | 901; <0.001*** | 0.83 (0.24) | 102 (0.4) | 534.5; 0.006** | 0.60 (0.13) | 0.74 (0.42) | 278; 0.02* |
| DLS | 0.35 (0.08) | 0.37 (0.1) | 653; 0.44 | 0.38 (0.06) | 0.39 (0.05) | 446; 0.19 | 0.36 (0.05) | 0.40 (0.08) | 232; 0.24 |
| ASYM | 0.02 (0.06) | 0.03 (0.03) | 666; 0.36 | 0.02 (0.05) | 0.03 (0.03) | 740; 0.50 | 0.05 (0.06) | 0.04 (0.03) | 155; 0.41 |
| STV | 0.004 (0.05) | 0.05 (0.06) | 835; 0.004** | 0.04 (0.02 | 0.07 (0.05) | 575; <0.001*** | 0.02 (0.03) | 0.06 (0.04) | 297; 0.02* |
| DTC_Walking_ Number of Steps [%] (n=44) |  |  |  |  |  |  | 7.69 (14.3) | 6.45 (17.7) | 135; 0.21 |
| DTC_Walking_ gait speed [%] (n=44) |  |  |  |  |  |  | 17 (23.5) | 5.42 (25.2) | 140 (42) 0.27 |
| DTC_Walking_ DLS [%] (n=44) |  |  |  |  |  |  | 9.26 (10.2) | 0.95 (19.4) | 118 (42) 0.09 |
| DTC_Walking_ ASYM [%] (n=44) |  |  |  |  |  |  | 78.1 (83.3) | 7.44 (120) | 69 (42) 0.003** |
| DTC_Walking_ STV [%] (n=44) |  |  |  |  |  |  | 106 (114) | -3.88 (106) | 45 (42) <0.001*** |

^a^, asymptotic p-value for Mann-Whitney-U-test (level of significance α≤0.05); ASYM, asymmetry; ^b^, p-value for Fisher’s exact test (level of significance α≤0.05); DLS, double limb support; DT, dual task; DTC_Walking_, dual task costs for walking while doing a second task (in percentage, %); IQR, interquartile range; LEDD, levodopa equivalence daily dose (in milligram, mg); MDS-UPDRS III, Movement Disorder Society-revised version of the motor part of the Unified Parkinson's Disease Rating Scale; n, sample size; s, seconds; ST, single task; STV, step time variability; W, test statistic for Mann-Whitney-U-test; ΔTMT, delta of Trail Making Test (part B minus part A); p≤0.05*, significant on level of significance α≤0.05; p≤0.01**, significant on level of significance p≤0.001***, significant on level of significance α≤0.001.
